# Adaptive QM/MM for Molecular Dynamics Simulations: 5. On the Energy-Conserved Permuted Adaptive-Partitioning Schemes

**DOI:** 10.3390/molecules23092170

**Published:** 2018-08-28

**Authors:** Adam W. Duster, Chun-Hung Wang, Hai Lin

**Affiliations:** Department of Chemistry, University of Colorado Denver, Denver, CO 80217, USA; ADAM.DUSTER@ucdenver.edu (A.W.D.); nakada0155@gmail.com (C.-H.W.)

**Keywords:** combined QM/MM, molecular dynamics simulations, energy conservation, radial distribution function, adaptive partitioning

## Abstract

In combined quantum-mechanical/molecular-mechanical (QM/MM) dynamics simulations, the adaptive-partitioning (AP) schemes reclassify atoms on-the-fly as QM or MM in a smooth manner. This yields a mobile QM subsystem with contents that are continuously updated as needed. Here, we tailor the Hamiltonian adaptive many-body correction (HAMBC) proposed by Boreboom et al. [*J. Chem. Theory Comput.* **2016**, *12*, 3441] to the permuted AP (PAP) scheme. The treatments lead to the HAMBC-PAP method (HPAP), which both conserves energy and produces accurate solvation structures in the test of “water-in-water” model system.

## 1. Introduction

Molecular dynamics (MD) simulations of diffusive systems, such as the diffusion of a solute (a solvated ion or molecule) through solvent, has been a challenging task for multiscale methods, especially for combined quantum-mechanics/molecular-mechanics (QM/MM) methods [1,2,3,4,5,6,7,8,9,10,11,12,13,14,15,16,17,18,19,20,21,22,23,24,25,26]. In conventional QM/MM methodology, atoms are designated as QM or MM at the beginning of a simulation and do not change these identities throughout a simulation. This causes difficulties when solvent molecules are exchanged between the solute’s solvation shells and the bulk solution, which may occur frequently. Adaptive QM/MM mitigates these problems by reclassifying the atoms as QM or MM on-the-fly based on their positions, assuring that the solute and its solvation shells are always described at the QM level of theory [25,27,28,29,30,31,32,33,34,35,36,37,38,39,40,41,42,43,44,45,46,47,48,49,50,51,52]. As a result, the QM and MM partitions in adaptive QM/MM are dynamically updated as needed, in contrast to the static partitions in conventional QM/MM.

**Figure 1:**
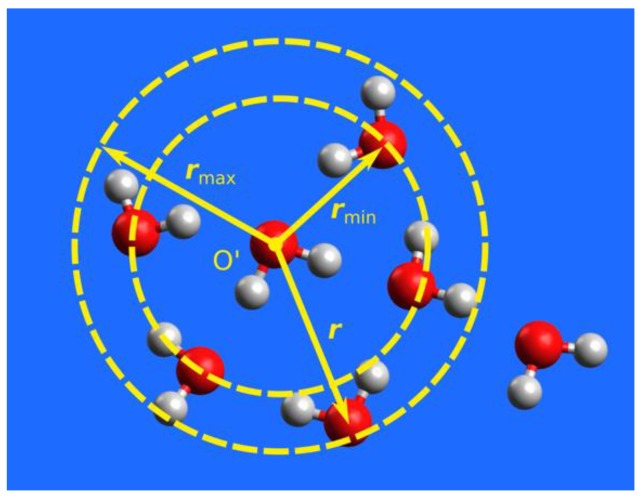
Adaptive QM/MM exemplified by a “water-in-water” model. One selected water molecule (oxygen atom labeled by O′) serves as the active-zone center. This molecule and its immediate surrounding water molecules within a distance ($r<r_{\min}$) are treated at the QM level of theory, while those at remote distances ($r>r_{\max}$) at the MM level. The water molecules at intermediate distances ($r_{\max}\geq r\geq r_{\min}$) are in the buffer zone and have mixed QM and MM characters. The energy of the system and the gradients of all (QM, buffer, and MM) atoms are smoothly interpolated when molecules migrate into, across, or out of the buffer zone.

One adaptive QM/MM algorithm is the permuted adaptive partitioning (PAP) scheme [29,32], which uses distance-based criteria for the QM and MM partitioning (Figure 1). In PAP, the QM zone, also called the active zone, consists of the solute and all molecules within a preset cutoff distance $r_{\min}$ from the solute. A group-based prescription is often adopted, where a whole molecule is treated as an entity, and the distance from the solute r is measured using the center of mass or a representative atom of the entire molecule. The description for whole molecules can also be applied to molecular fragments, such as functional groups [32]. The MM zone, also known as the environmental zone, consists of molecules with $r>r_{\max}$, where $r_{\max}$ is another preset cutoff distance. Between the QM and MM zones is the buffer zone ($r_{\max}\geq r\geq r_{\min}$), and the molecules in the buffer zone are often called the buffer groups. The energy of the system and the gradients of all (QM, buffer, and MM) atoms are smoothly interpolated when molecules or functional groups migrate into, across, or out of the buffer zone. This is accomplished by expressing the QM/MM potential as a weighted sum of many-body contributions that vary continuously and smoothly as the buffer groups change their positions. The PAP method conserves energy and momentum, and it has been found to yield superior numerical stabilities in MD simulations [29,32].

A challenge in PAP (and other distance-based adaptive QM/MM methods) concerns the gradients due to the smoothing functions employed in the interpolation procedure, which, if not negligible, may cause artefacts in the MD simulations [30,35,42,46]. These forces, which are sometimes called transition forces, are proportional to the difference between the QM and MM energies at the current geometry. More specifically, the energy difference is the energy released (or absorbed) when a buffer group is switched from MM to QM while holding the QM or MM classifications of the other groups as well as all atomic coordinates fixed [42]. These transition forces are therefore associated with the difference in chemical potential between the QM and MM descriptions of the given buffer group. These transition forces drive the molecules moving towards the QM or MM zones, even in the absence of the interpolated gradients between the QM and MM potential derivatives, which are considered the “real” or “physical” forces.

In principle, the effects due to these transition forces can be eliminated or minimized by carefully aligning the QM and MM potentials [29]. However, it is difficult to align multi-dimensional potentials, and a simple and general algorithm to for potential alignment has not yet been developed. An alternative solution is the modified PAP (mPAP) scheme, where external forces are applied to cancel out these artificial forcs [35]. Mathematically, the transition forces are simply deleted. Conceptually, this means that the chemical potentials are equalized at every step for the system [46]. It has been demonstrated that mPAP yields reasonably accurate structures and dynamics in MD simulations [35,40,43]. The downside is that, because of the involvement of the external forces, the scheme no longer has a Hamiltonian formulation and therefore cannot be used for studies where Hamiltonian systems are required [43]. 

Recently, Boreboom et al. [42] proposed the Hamiltonian adaptive many-body correction (HAMBC) for sorted adaptive-partitioning (SAP) QM/MM simulations. Inspired by the works of the molecular-mechanics/course-grained (MM/CG) community, especially the Hamiltonian adaptive resolution scheme (H-AdResS) by Potestio et al. [53] the HAMBC method includes per-molecule-based correction terms to the SAP QM/MM Hamiltonian. By design, the gradients of the correction terms should cancel out the *average* transition forces due to the smoothing functions; the cancellation is not necessarily exact at any single time step. The HAMBC was demonstrated in test calculations of a “water-in-water” model using dual MM levels, where a selected water molecule as the solute and its solvation shell are treated by one MM force field model and the bulk solvent by another MM force field model [42]. (Due to their high efficiencies, dual-MM test calculations have frequently been employed in testing adaptive QM/MM schemes, e.g., in the development of the original PAP scheme [29].) It is encouraging to find that HAMBC was able to produce correct solvation structures for the selected solute water while conserving energy in SAP simulations [42]. 

In this paper, we report the tailoring of the HAMBC treatment to the much more sophisticated PAP method. In the HAMBC by Boreboom et al. [42] the per-molecule correction term is a function of the fractional “QM character” for a solvent molecule, which is the sum of the weights of the contributing partitions that describe this solvent molecule at the QM level. Because in general there is no analytical function for the correction term, the correction must be calculated through thermodynamic integration over the selected variable. In PAP, it is more convenient to use the value of the smoothing function than the QM character for the given buffer group. We have previously shown that this QM character is equivalent to the value of the smoothing function for a given buffer group in a fully expanded PAP potential [46]. However, the many-body expansion of the PAP potential is often truncated to reduce computational costs, and in a truncated PAP potential, the value of the smoothing function no longer equals the QM character. In this work, we demonstrate that the correction can be taken as a function of the value of the smoothing function even when the PAP potential is truncated. 

## 2. Materials and Methods 

### 2.1. The PAP Algorithm 

In PAP, the QM/MM potential is expressed using a many-body expansion: [29]

(1a) $$\begin{array}{l} V=V^{A}+\sum_{i=1}^{N}P_{i}\left(V_{i}^{A}-V^{A}\right)+\sum_{i=1}^{N-1}\sum_{j=i+1}^{N}P_{i}P_{j}\left(V_{i,j}^{A}-\left(V^{A}+\sum_{r=i,j}^{}\left(V_{r}^{A}-V^{A}\right)\right)\right)\\ \;+\sum_{i=1}^{N-2}\sum_{j=i+1}^{N-1}\sum_{k=j+1}^{N}P_{i}P_{j}P_{k}(V_{i,j,k}^{\text{A}}-(V^{\text{A}}+\sum_{r=i,j,k}\left(V_{r}^{\text{A}}-V^{\text{A}}\right)\\ \;+\sum_{\left(p,q\right)=\left(i,j\right),\left(i,k\right),\left(j,k\right)}\left(V_{p,q}^{\text{A}}-\left(V^{\text{A}}+\sum_{r=p,q}^{}\left(V_{r}^{\text{A}}-V^{\text{A}}\right)\right)\right)))+\cdots \end{array}$$

Or more compactly, [32]

(1b) $$V=V^{A}\overset{N}{\underset{i=1}{\prod }}\left(1-P_{i}\right)+\overset{N}{\underset{i=1}{\sum }}V_{i}^{A}P_{i}\overset{N}{\underset{j\;\neq \;i}{\prod }}\left(1-P_{j}\right)+\overset{N-1}{\underset{i=1}{\sum }}\overset{N}{\underset{j=i+1}{\sum }}V_{i,j}^{A}P_{i}P_{j}\overset{N}{\underset{k\;\neq \;j\;\neq \;i}{\prod }}\left(1-P_{k}\right)+\cdots$$

(1c) $$=V^{A}\sigma_{0}+\overset{N}{\underset{i=1}{\sum }}V_{i}^{A}\sigma_{i}+\overset{N-1}{\underset{i=1}{\sum }}\overset{N}{\underset{j=i+1}{\sum }}V_{i,j}^{A}\sigma_{i,j}+\cdots$$

Here, $V^{A}$ is the QM/MM energy of the partitions with no buffer group treated at the QM level, $V_{i}^{A}$ with the *i*-th buffer group treated at the QM level, $V_{i,j}^{A}$ with the *i*-th and *j*-th buffer groups treated at the QM level, … $V_{1,2,\cdots ,N}^{A}$ with all *N* buffer groups at the QM level, $P_{i}$ is the smoothing function for the *i*-th buffer group, and the weights $\sigma_{0}$, $\sigma_{i}$, $\sigma_{i,j}$… are assigned to the partitions $V^{A}$, $V_{i}^{A}$, $V_{i,j}^{A}$… respectively. Note that *N* can vary from one time step to another. We have chosen the smoothing function $P_{i}$ to be a fifth-order spline:

(2) $$P\left(\alpha_{i}\right)=\{\begin{array}{c} \begin{array}{cc} 0 & \alpha_{i}>1 \end{array}\\ \begin{array}{cc} -6\alpha_{i}{}^{5}+15\alpha_{i}{}^{4}-10\alpha_{i}{}^{3}+1,\; & 1\geq \alpha_{i}\geq 0 \end{array}\\ \begin{array}{cc} 1, & \alpha_{i}<0 \end{array} \end{array}$$

where $\alpha_{i}$ is a reduced distance for the *i*-th buffer group,

(3) $$\alpha_{i}=\frac{r_{i}-r_{\min}}{r_{\max}-r_{\min}}$$

where the distance $r_{i}=\left|r_{i}\right|=\left|x_{i}-x_{A}\right|$ is between the buffer group at position $x_{i}$ and the solute at position $x_{A}$, and $r_{\max}\geq r_{i}\geq r_{\min}$. The smoothing function $P_{i}$ is continuous and differentiable over the domain [0, 1], and corresponds to all possible $r_{i}$ that a buffer group can take in the buffer zone. The PAP scheme is a Hamiltonian algorithm and conserves energy.

According to Equation (1), a buffer group is treated by QM in some partitions and by MM in the others, in line with its dual QM-MM characteristics. This contrasts with the groups in the QM and MM zones, which remain as QM and MM, respectively, in all possible partitions. (Note that the QM and MM zones are also called the active and environmental zones, respectively.) All derivatives of the PAP potential energy with respect to the coordinates vary smoothly up to the same order for which $P_{i}$ varies continuously. The full expansion of the PAP potential requires $2^{N}$ calculations. However, the potential is typically truncated and only the terms from a subset of lower-orders are calculated to increase computational efficiency. 

If we denote the included partitions by *S*, be it a subset or the total of all the partitions at a given time step, the PAP potential can be written as:

(4) $$V=\underset{s\in S}{\sum }V_{s}\sigma_{s}$$

The derivative with respect to any atomic Cartesian coordinate component $x_{t}$ is:

(5) $$-\frac{\partial V}{\partial x_{t}}=\underset{s\in S}{\sum }\left(-\sigma_{s}\frac{\partial V}{\partial x_{t}}-V_{s}\frac{\partial \sigma_{s}}{\partial x_{t}}\right)$$

The first term ($F_{\mathrm{phys}}=-\sum_{s\in S}\sigma_{s}\frac{\partial V}{\partial x_{t}}$), the negative of the sum of the smoothed gradients, represents the interpolated physical forces between the QM and MM forces. The second term ($F_{\mathrm{trans}}=-\sum_{s\in S}V_{s}\frac{\partial \sigma_{s}}{\partial x_{t}}$) corresponds to the gradients due to the smoothing functions and is the so-called transition forces. The transition forces are associated with the difference in chemical potentials between the QM and MM regions [42]. These transition forces are pairwise, acting on both the solute molecule at the QM-zone center and the buffer groups, and they cause structural artifacts if non-negligible. This “transition-force problem” is universal in distance-based adaptive QM/MM algorithms and is not limited to PAP [46]. Various treatments have been implemented to eliminate the problem or minimize its effects. For example, in the non-Hamiltonian mPAP method [35], these transition forces are deleted, and as a result, the remaining forces acting on the atoms no longer correspond to the potential defined in Equation (1). The mPAP scheme has been shown to yield very accurate structural and dynamics properties in a number of tests [35,40,41]. 

### 2.2. HAMBC Expression for PAP

To minimize the effects due to the transition forces while maintaining energy conservation, Boreboom et al. [42] recently proposed the HAMBC for SAP QM/MM, which also suffers from the transition force problem. In HAMBC-SAP, an energy correction term is added to the SAP potential for every buffer group. The correction term is assumed to depend only on the type of the buffer group (e.g., water versus ethanol) and the so-called “QM character,” which is the sum of the weights of the contributing partitions that describe this buffer group at the QM level. For PAP, it would be more convenient to select the smoothing function $P_{i}$ than to choose the QM character. Both $P_{i}$ and the QM character are metrics related to the buffer group′s position within the buffer zone; the QM character is equivalent to $P_{i}$ in a fully expanded PAP potential but differs otherwise [46]. The theory and implementations for HAMBC-PAP (HPAP for short) are described below.

#### 2.2.1. A Mean-Field Treatment of the Individual Group Corrections

As in Ref. [42], we assume here that the total correction is a sum of individual group corrections over all *N* buffer groups and that the individual *i*-th group correction ($H_{i}^{C}\left(r_{i}\right)$) only depends on the type of species of the group and on the group′s distance $r_{i}$ to the active-zone center. Because $P_{i}$ decreases monotonously from 1 to 0 as $r_{i}$ increases from $r_{\min}$ to $r_{\max}$, the correction term $H_{i}^{C}\left(r_{i}\right)$ can also be expressed as a function of the dimensionless $P_{i}$, i.e., $H_{i}^{C}\left(P_{i}\right)$. That the corrections to the buffer groups are independent of each other implies that the interactions between the groups are treated in a “mean-field” manner as the correlations between the groups are neglected. Thus, many-body effects are treated in an average fashion. For simplicity, we only consider one species, the solvent. Under this circumstance, the new HPAP potential is:

(6) $$V^{\mathrm{HPAP}}=\underset{s\in S}{\sum }V_{s}\sigma_{s}-\overset{N}{\underset{i=1}{\sum }}H_{i}^{C}\left(P_{i}\right)=\underset{s\in S}{\sum }V_{s}\sigma_{s}-\overset{N}{\underset{i=1}{\sum }}H^{C}\left(P_{i}\right)$$

The subscript *i* in $H_{i}^{C}\left(P_{i}\right)$ is dropped because all corrections are identical for the same species.

The corresponding forces are given by:

(7) $$-\frac{\partial V^{\mathrm{HPAP}}}{\partial x_{t}}=-\underset{s\in S}{\sum }\left(\sigma_{s}\frac{\partial V}{\partial x_{t}}+V_{s}\overset{N}{\underset{i=1}{\sum }}\frac{\partial \sigma_{s}}{\partial P_{i}}\frac{dP_{i}}{dr_{i}}\frac{\partial r_{i}}{\partial x_{t}}\right)+\overset{N}{\underset{i=1}{\sum }}\frac{dH^{c}\left(P_{i}\right)}{dP_{i}}\frac{dP_{i}}{dr_{i}}\frac{\partial r_{i}}{\partial x_{t}}$$

The correction force due to the derivative of the individual correction energy ($F_{\mathrm{corr}}=-\frac{dH^{c}\left(P_{i}\right)}{dP_{i}}\frac{dP_{i}}{dr_{i}}\frac{\partial r_{i}}{\partial x_{t}}$) is designed such that, at any given distance $r_{i}$ that separates the solute molecule and the *i*-th buffer group, it should cancel the average transition forces acting on the buffer group:

(8a) $$\langle -\underset{s\in S\;}{\sum }V_{s}\frac{\partial \sigma_{s}}{\partial P_{i}}\frac{dP_{i}}{dr_{i}}\frac{\partial r_{i}}{\partial x_{t}}\rangle +\frac{dH^{c}\left(P_{i}\right)}{dP_{i}}\frac{dP_{i}}{dr_{i}}\frac{\partial r_{i}}{\partial x_{t}}=0$$

The requirement is enforced for any given $r_{i}$ and $x_{t}$. Therefore, if follows that

(8b) $$\langle -\underset{s\in S\;}{\sum }V_{s}\frac{\partial \sigma_{s}}{\partial P_{i}}\rangle +\frac{dH^{c}\left(P_{i}\right)}{dP_{i}}=0$$

The relevant $x_{t}$ are the coordinates of the buffer group. Based on Newton’s Third Law of Motion, such cancellation will automatically be realized at the active-zone center. 

We compute the individual correction derivative $\frac{dH^{c}\left(P_{i}\right)}{dP_{i}}$ as the average energy difference between a pair of partitions where the *only* variation is that the *i*-th group at $r_{i}$ is treated at QM or MM:

(9) $$\frac{dH^{c}\left(P_{i}\right)}{dP_{i}}=\langle V_{i=\mathrm{QM}}\left(r_{i}\right)-V_{i=\mathrm{MM}}\left(r_{i}\right)\rangle =\langle \Delta E_{i}\left(r_{i}\right)\rangle =\langle \Delta E_{i}\left(P_{i}\right)\rangle$$

Here, the energies of the partition pair are denoted $V_{i=\mathrm{QM}}\left(r_{i}\right)$ and $V_{i=\mathrm{MM}}\left(r_{i}\right)$, respectively, and 〈⋯〉 indicates the corresponding average. As usual, the zeros of the potentials $V_{i=\mathrm{QM}}$ and $V_{i=\mathrm{MM}}$ are chosen such that these energies represent the net interaction energies [46]. At any given time step of the simulation, the number of partitions depends on the truncation order of mPAP potential, and usually there are multiple such pairs of partitions for the *i*-th group at $r_{i}$, each with different buffer groups treated by QM. Thus, the average is over the partition pairs for the buffer group at $r_{i}$ in a sampled geometry and over all sampled geometries while keeping the buffer group at $r_{i}$ in the mPAP simulations. By varying $r_{i}$ continuously, one obtains the complete profile of $\langle \Delta E_{i}\rangle$. Again, because $P_{i}$ decreases monotonously from 1 to 0 as $r_{i}$ increases from $r_{\min}$ to $r_{\max}$, the average energy difference $\langle \Delta E_{i}\rangle$, which depends solely on $r_{i}$, can also be expressed as a function of the dimensionless $P_{i}$. 

#### 2.2.2. Calculations of $\langle \Delta E_{i}\left(P_{i}\right)\rangle$ and $H^{C}\left(P_{i}\right)$

First, we consider one partition pair for a sampled geometry at a given time step. Let us assume that the partition of $V_{i=\mathrm{QM}}\left(r_{i}\right)$ has a QM subsystem consists of the active-zone “A”, the *i*-th group, and (m−1) other groups selected from the *N* buffer groups. Accordingly, the other partition of $V_{i=\mathrm{MM}}\left(r_{i}\right)$ will feature a QM subsystem consisting of the active-zone “A” and the other (m−1) buffer groups, since the *i*-th group is treated by MM. These two partitions correspond to the *m*-th and (m−1)-th order terms in the mPAP potential, respectively. For the sake of brevity, we refer to this partition pair as of the *m*-th order. Without losing generality, we label these *m* buffer groups by 1, 2 … *m*. The energy difference for this partition pair is

(10) $$\Delta E_{i}^{\left(m\right)}\left(P_{i}\right)=\Delta E_{i}^{\left(m\right)}\left(r_{i}\right)=V_{1,2,\cdots ,i-1,i,i+1,\cdots ,m}^{A}\left(r_{i}\right)-V_{1,2,\cdots ,i-1,i+1,\cdots ,m}^{A}\left(r_{i}\right)$$

Next, we combine all such partition pairs corresponding to a given *m*-th order term in the mPAP Hamiltonian, where there are $\left(\begin{array}{c} N-1\\ m-1 \end{array}\right)=\frac{\left(N-1\right)!}{\left(m-1\right)!\left(N-m\right)!}$ such pairs, up to the *p*-th order at which the mPAP Hamiltonian is truncated, all for the sampled geometry at the given time step. That is, we take the average of $\Delta E_{i}^{\left(m\right)}$ over partition pairs for a given *m* and then over *m* for m=1, 2,…,p. This gives the average energy difference for a given sampled geometry:

(11) $$\bar{\Delta E_{i}}=\frac{\Delta E_{i}^{\left(1\right)}+\sum_{\mathrm{all}\;\mathrm{pairs}}^{}\Delta E_{i}^{\left(2\right)}+\sum_{\mathrm{all}\;\mathrm{pairs}}^{}\Delta E_{i}^{\left(3\right)}+\cdots }{\left(\begin{array}{c} N-1\\ 0 \end{array}\right)+\left(\begin{array}{c} N-1\\ 1 \end{array}\right)+\left(\begin{array}{c} N-1\\ 2 \end{array}\right)+\cdots }$$

We then average $\bar{\Delta E_{i}}$ over all sampled geometries in the simulations where the *i*-th group is located at $r_{i}$. This gives $\langle \Delta E_{i}\rangle$ for the corresponding $P_{i}$. The complete curve of $\langle \Delta E_{i}\left(P_{i}\right)\rangle$ is obtained by varying $P_{i}$ continuously from 0 to 1. For better statistics, we also average over all buffer groups of the same type in the simulations. 

In general, there is no analytic form for $H^{C}\left(P_{i}\right)$, which must be obtained through thermodynamic integration:

(12) $$H^{C}\left(P_{i}\right)=\int_{0}^{P_{i}}\langle \Delta E_{i}\left(P_{i}^{\prime }\right)\rangle dP_{i}^{\prime }$$

where $P_{i}^{\prime }$ is the dummy variable for integration. 

The final correction is applied to each group in the system according to the following scheme:

(13) $$H^{C}\left(P_{i}\right)=\{\begin{array}{c} \begin{array}{cc} H^{C}\left(0\right), & r_{i}>r_{\max} \end{array}\\ \begin{array}{cc} H^{C}\left(P_{i}\right),\; & r_{\max}\geq r_{i}\geq r_{\min} \end{array}\\ \begin{array}{cc} H^{C}\left(1\right), & r_{i}<r_{\min} \end{array} \end{array}$$

We note that $H^{C}\left(0\right)\neq H^{C}\left(1\right)$ in general. 

#### 2.2.3. Many-Body Contributions to $\Delta E_{i}^{\left(m\right)}$

To see how many-body interactions are incorporated into the energy difference for a partition pair, we use the standard formula of many-body expansion for a system made of *n* monomers:

(14) $$V_{1,2,\cdots ,n}=\overset{n}{\underset{a=1}{\sum }}\varepsilon_{a}+\overset{n-1}{\underset{a=1}{\sum }}\overset{n}{\underset{b=a+1}{\sum }}\varepsilon_{ab}+\overset{n-2}{\underset{a=1}{\sum }}\overset{n-1}{\underset{b=a+1}{\sum }}\overset{n}{\underset{c=b+1}{\sum }}\varepsilon_{abc}+\cdots$$

where $\varepsilon_{a}$ is the energy of an isolated monomer, $\varepsilon_{ab}$ is the energy of a dimer minus the energies of the monomers it comprises (i.e., it is the pairwise interaction energy), etc. Applying Equation (14) to $V_{1,2,\cdots ,i-1,i,i+1,\cdots ,m}^{A}\left(r_{i}\right)$ and $V_{1,2,\cdots ,i-1,i+1,\cdots ,m}^{A}\left(r_{i}\right)$, and after canceling identical terms, one has

(15) $$\Delta E_{i}^{\left(m\right)}=\left(\Delta \varepsilon_{i}+\Delta \varepsilon_{Ai}\right)+\overset{m}{\underset{j\;\neq \;i}{\sum }}\left(\Delta \varepsilon_{ji}+\Delta \varepsilon_{Aji}\right)+\overset{m}{\underset{j\;\neq \;i}{\sum }}\overset{m}{\underset{k\;\neq \;j\;\neq \;i}{\sum }}\left(\Delta \varepsilon_{jki}+\Delta \varepsilon_{Ajki}\right)+\cdots$$

where

(16a) $$\Delta \varepsilon_{i}=\varepsilon_{i=\mathrm{QM}}-\varepsilon_{i=\mathrm{MM}}$$

(16b) $$\Delta \varepsilon_{Ai}=\varepsilon_{A,i=\mathrm{QM}}-\varepsilon_{A=\mathrm{QM};\;i=\mathrm{MM}}$$

(16c) $$\Delta \varepsilon_{ji}=\varepsilon_{j,i=\mathrm{QM}}-\varepsilon_{j=\mathrm{QM};\;i=\mathrm{MM}}$$

(16d) $$\Delta \varepsilon_{Aji}=\varepsilon_{A,j,i=\mathrm{QM}}-\varepsilon_{A,k=\mathrm{QM};\;i=\mathrm{MM}}$$

(16e) $$\Delta \varepsilon_{jki}=\varepsilon_{j,k,i=\mathrm{QM}}-\varepsilon_{j,k=\mathrm{QM};\;i=\mathrm{MM}}$$

(16f) $$\begin{array}{l} \Delta \varepsilon_{Ajki\;}=\varepsilon_{A,j,k,i=\mathrm{QM}}-\varepsilon_{A,j,k=\mathrm{QM};\;i=\mathrm{MM}}\\ \ldots \end{array}$$

Here $\varepsilon_{i=\mathrm{QM}}$ and $\varepsilon_{i=\mathrm{MM}}$ are the QM and MM energies of the *i*-th group at the QM and MM levels, respectively; $\varepsilon_{j,i=\mathrm{QM}}$ is the pairwise interaction energy between groups *i* and *j*, both treated at the QM level; $\varepsilon_{j=\mathrm{QM};\;i=\mathrm{MM}}$ is the pairwise interaction energy between group *j* described at the QM level and group *i* at the MM level; $\varepsilon_{j,k,i=\mathrm{QM}}$ is the three-body contribution by groups *i*, *j*, and *k* treated at the QM level; $\varepsilon_{j,k=\mathrm{QM};i=\mathrm{MM}}$ is the three-body contribution by groups *j* and *k* described at the QM level and group *i* at the MM level… Terms involving the active-zone “A” are defined similarly. The many-body interactions where the *i*-th group treated by MM depend on the specific QM/MM embedding model. Note that these many-body interactions never need to be explicitly evaluated; they are prescribed here merely to assist understanding of the many-body interactions in $\Delta E_{i}^{\left(m\right)}$. The above analysis indicates that $\Delta E_{i}^{\left(m\right)}$ is a sum of the differences between the many-body terms in the partition pair involving the *i*-th group in the many-body expansions up to the *m*-th order.

#### 2.2.4. Effects Due to Truncation in mPAP Hamiltonian

If the mPAP potential is truncated at a low order *p* and if N≫p, which is the case in our current simulations, the average is dominated by the *p*-th order $\Delta E_{i}^{\left(p\right)}$, which has the largest number of partitions:

(17) $$\langle \Delta E_{i}\rangle \approx \langle \Delta E_{i}^{\left(p\right)}\rangle$$

Moreover, because all groups are the same type of molecules,

(18) $$\begin{array}{l} \langle \Delta E_{i}^{\left(p\right)}\rangle =\left(\langle \Delta \varepsilon_{i}\rangle +\langle \Delta \varepsilon_{Ai}\rangle \right)+\left(p-1\right)\left(\langle \Delta \varepsilon_{ji}\rangle +\langle \Delta \varepsilon_{Aji}\rangle \right)\\ \;+\frac{\left(p-1\right)\left(p-2\right)}{2}\left(\langle \Delta \varepsilon_{jki}\rangle +\langle \Delta \varepsilon_{Ajki}\rangle \right)+\cdots \end{array}$$

That is, $\langle \Delta E_{i}\rangle$ depends on the truncation order in the mPAP potential. A special case is dual-MM, where effective two-body potentials are employed, the third and higher order terms are 0 in Equation (14). Consequently, $\langle \Delta \varepsilon_{Aji}\rangle$, $\langle \Delta \varepsilon_{jki}\rangle$, and higher-order contributions vanish, leading to

(19) $$\langle \Delta E_{i}^{\left(p\right)}\rangle =\left(\langle \Delta \varepsilon_{i}\rangle +\langle \Delta \varepsilon_{Ai}\rangle \right)+\left(p-1\right)\langle \Delta \varepsilon_{ji}\rangle$$

### 2.3. Simulation Details 

An Open-MP-based parallel version of HPAP method has been implemented in our in-house code, QMMM [54]. The QMMM code calls an MM program such as NAMD [55] for MM calculations and a QM program such as MNDO [56] for QM calculations, synthesizes the energies and gradients from both, and propagates the trajectory. Here, to reduce the computational expense from QM/MM calculations, we carry out dual-MM (MM′/MM) simulations, as we have done previously in the development of the original PAP algorithm [29] and as Boreboom et al. [42] did in their HAMBC study. Since we are only focusing on the adaptive-boundary treatments, the use of dual-MM test calculations suffices. 

The model system for all calculations is a 30.25 × 30.25 × 30.25 Å^3^ water box that contains 915 water molecules (density = 0.99 g/mL). The water box was prepared by equilibration for 10 ns under the *NVT* ensemble at the single-MM level using the TIP3P/Fs [57,58] water model. For the adaptive-partitioning simulations, each water molecule is an adaptive partitioning group, with the oxygen atom designated as the representative atom for the group. One water oxygen was chosen to be the active-zone center for the simulation, and the buffer and active zone radii were set to $r_{\max}=6.4$ Å and $r_{\min}=5.5$ Å respectively. All adaptive-partitioning potentials were truncated at the 2nd order. For all calculations, periodic boundary conditions with the minimum-image convention were adopted. The cutoff for non-bonded interactions was set to 12 Å with smoothing switched on at 11 Å. All simulations are under constant volume with a time step of 0.5 fs. If constant temperatures were required, a Nosé-Hoover [59] thermostat of 298.15 K was coupled to the model system with a coupling coefficient of 40 fs. 

**Figure 2:**
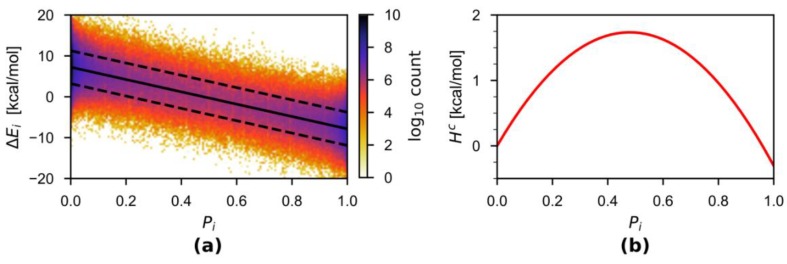
(**a**) Density plot of $\Delta E\left(P_{i}\right)$ computed from the combined 50-ps simulations. The solid and dashed black lines indicated the $\langle \Delta E\left(P_{i}\right)\rangle$ and the associated standard deviation, respectively. Linear regression yielded an analytical representation for $\langle \Delta E\left(P_{i}\right)\rangle =\left(-15.087P_{i}+7.236\right)$ kcal·mol^−^^1^ with *R*^2^ = 0.9997. The average standard deviation over the 100 bins is 4.045 kcal·mol^−^^1^. (**b**) The energy correction term $H^{C}\left(P_{i}\right)$ computed by integration using the analytical representation of $\langle \Delta E\left(P_{i}\right)\rangle$.

First, to obtain the correction term $H^{C}$, the model was simulated at constant temperature at the MM′/MM level using the mPAP scheme, where MM′ = SPC/Fw [58] and MM = TIP3P/Fs [57,58] (the force field parameters are compared in Table 1). Five 20-ps trajectories were propagated. Only the second half 10 ps of every trajectory was utilized to determine the correction term $H^{C}$, during which the energies of the partitions were recorded. Note that multiple partitions and thus multiple energy differences are available at one single time step, unless there is no or only one buffer group. The combined 50-ps trajectories for the $H^{C}$ calculations resulted in a total of 16,791,809 energy differences ($\Delta E_{i}^{\left(1\right)}$ and $\Delta E_{i}^{\left(2\right)}$). Figure 2 shows the density distribution of $\Delta E_{i}\left(P_{i}\right)$. We found that the 50 ps combined simulation time was sufficient to converge the correction term in the present work (more details in the Results section). These energy differences $\Delta E_{i}\left(P_{i}\right)$ were added to 100 bins equally spaced over the domain [0, 1] according to the $P_{i}$ value of the buffer group. The average of each bin was then taken and used as input for linear regression to obtain the function $\Delta E\left(P_{i}\right)$. We have dropped the subscript *i*, because $\Delta E_{i}\left(P_{i}\right)$ is identical for all buffer groups here. The linear regression was motivated by the approximately linear nature of the $\Delta E\left(P_{i}\right)$ curve (more details in the Discussion section). The analytical integral of the regression line was used to obtain $H^{C}\left(P_{i}\right)$. Both $\Delta E\left(P_{i}\right)$ and $H^{C}\left(P_{i}\right)$ were discretized into 10,000 evenly spaced points between the domain [0, 1] and are stored as a look-up table read in by the QMMM source code. The correction for a group was chosen by looking up the $\Delta E\left(P_{i}\right)$ and $H^{C}\left(P_{i}\right)$ values in the table for the next largest $P_{i}$ value.

Second, to examine the solvation structures obtained through the HAMBC correction, we carried out adaptive-partitioning simulations at constant temperature using three schemes: PAP, mPAP, and HPAP. For each scheme, five trajectories were propagated using the final geometries and velocities from the five mPAP simulations used to compute $H^{C}$. Each trajectory was propagated for 10 ps. The combined 50 ps trajectories were employed to calculate the pairwise radial distribution function for a given scheme.

Finally, to examine the degree of energy conservation, we also performed *NVE* simulations for all three schemes. One trajectory for each method was propagated for 25 ps with 0.5 fs time steps.

## 3. Results

### 3.1. HAMBC Correction Term

Figure 2a plots the energy difference over the thermodynamic variable $\Delta E\left(P_{i}\right)$ obtained from the combined 50-ps trajectories, together with the linear regression results. The almost perfect linear fit is incidental due to the similarity in some parameters of the two employed force fields (see Section 4 for detailed discussion). In general, the linearity is very approximate or does not hold. Nevertheless, by taking advantage of the analytical representation of $\Delta E\left(P_{i}\right)$ from the linear regression, we estimated the correction term $H^{C}\left(P_{i}\right)$, which is depicted in Figure 2b.

**Figure 3:**
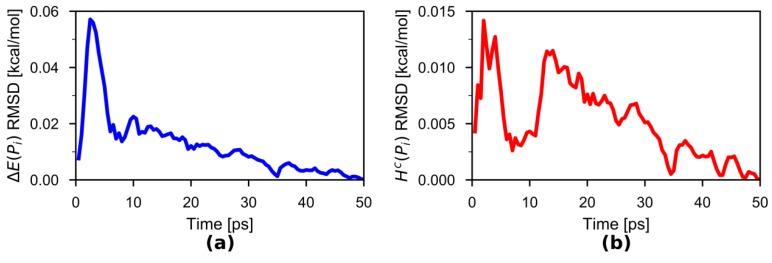
Root mean squared deviations (RMSDs) of (**a**) $\Delta E\left(P_{i}\right)$ and (**b**) $H^{C}\left(P_{i}\right)$, respectively, as functions of simulation time, taking their final curves from the combined 50-ps simulations as references.

To explore the length of simulation needed to generate the HPAP correction, we calculated the root mean squared deviation (RMSD) of $\Delta E\left(P_{i}\right)$ and $H^{C}\left(P_{i}\right)$ as functions of simulation time, taking as reference the final curves from the accumulated 50-ps simulations (Figure 3). The RMSD values of $\Delta E\left(P_{i}\right)$ and $H^{C}\left(P_{i}\right)$ were calculated over the 100 bins that are equally distributed bins along the $P_{i}$ domain [0, 1]. It can be seen that both RMSDs drop consistently after ~15 ps. The function $\Delta E\left(P_{i}\right)$ converged to below 0.01 kcal mol^−1^ after 30 ps of combined simulations. Because integration reduces the fluctuations in $\Delta E\left(P_{i}\right)$, the RMSD values of $H^{C}\left(P_{i}\right)$ are even smaller than those of $\Delta E\left(P_{i}\right)$.

**Figure 4:**
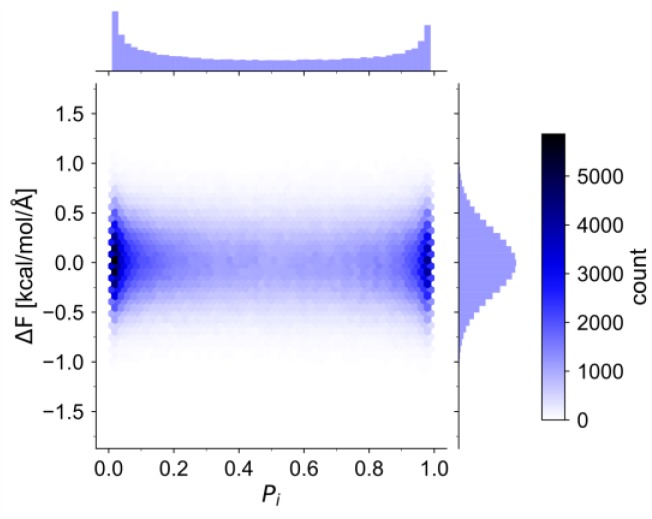
Bivariate distribution of the difference between the transition forces and the correction forces (defined as $\Delta F=F_{\mathrm{trans}}-F_{\mathrm{corr}}$) in the HPAP simulations.

A necessary requirement for the correction term is that the correction force $F_{\mathrm{corr}}$ must, on average, cancel out the transition force $F_{\mathrm{trans}}$. This fact is exemplified in the distribution of the difference between these two forces ($\Delta F=F_{\mathrm{trans}}-F_{\mathrm{corr}}$) in the final HPAP simulations (Figure 4). It can be seen that ΔF is distributed approximately normally around 0 kcal·mol^−1^Å^−1^ across the entire domain of $P_{i}$. The calculated average ΔF over all $P_{i}$ is 0.0007 kcal·mol^−1^Å^−1^, with a standard deviation of 0.4 kcal·mol^−1^Å^−1^, in excellent agreement with the cancellation requirement.

### 3.2. Solvation Structures 

**Figure 5:**
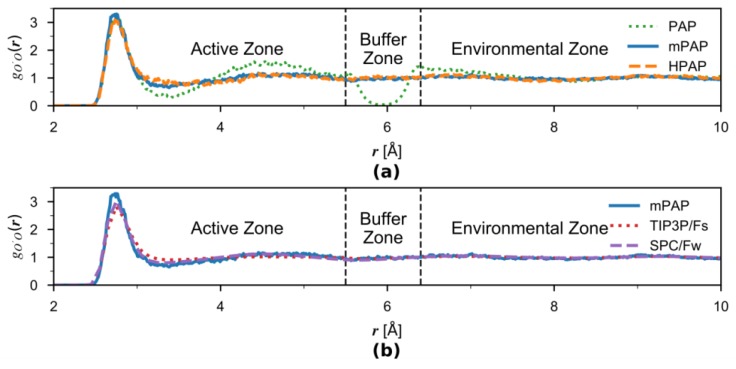
(**a**) Comparison of radial distribution functions (RDF) between AP simulations. The water oxygen atom O′ serves as the active-zone center, and O is the surrounding water oxygen atom. The results are illustrated by a green dotted line for the PAP, orange dashed line for the HPAP, and blue solid line for the reference mPAP methods, respectively. The buffer zone boundaries at $r_{\max}=6.4$ Å and $r_{\min}=5.5$ Å are indicated by the two black dashed vertical lines. (**b**) Comparisons of RDF between the mPAP and the two single-level MM simulations.

We examined the solvation structure around the solute water by computing the pairwise radial distribution function (RDF) $g_{O\text{’}O}\left(r\right)$ between its oxygen (O′, serving as the center of the active zone) and the surrounding oxygen (O) atoms for the original PAP, mPAP, and HPAP simulations under constant temperature (Figure 5a). The original PAP results show an erroneous dip around r=5.95 Å which corresponds to the center of the buffer zone. This is caused by the transition forces pushing water out of the buffer zone. However, this artifact is eliminated by inclusion of the correction term in HPAP, for which the RDF closely matches the mPAP reference data, indicating that the HPAP method is able to produce accurate solvation structures. The results here are in line with what were found by Boreboom et al. [42] although different adaptive schemes are employed. 

For comparison, we also present in Figure 5b the RDF computed from the mPAP and the two single-level MM simulations. All three RDF curves are overall quite similar, although the TIP3P/Fs water is slightly less structural than SPC/Fw. The mPAP RDF better resembles the SPC/Fw curve in the active-zone, as it is supposed to be. Interestingly, despite the similarity between SPC/Fw and TIP/Fs, a significantly distorted RDF was produced by PAP in the buffer-zone region, as observed in Figure 5a. This demonstrates the sensitivity of the RDF to the boundary treatment in the adaptive simulations and underscores the importance of proper treatments implemented in mPAP and HPAP.

**Figure 6:**
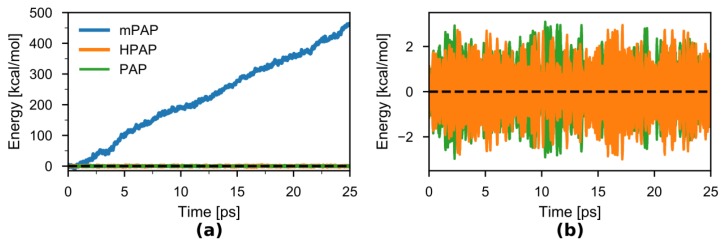
(**a**) Total energy as the sum of kinetic and potential energies of the model system in the 25-ps *NVE* simulations using the PAP (green), mPAP (blue), and HPAP (orange) methods. The many-body expansions of the potentials were all truncated at the 2nd order. (**b**) PAP and HPAP Total energies in greater detail. The zero of energy was chosen to be the mean energy of the first 250 fs.

Next, we checked the degree of energy conservation in the *NVE* simulations (Figure 6a,b). As expected, there was a large energy drift during the duration of the mPAP simulation of 17 kcal·mol^−1^·ps^−1^. On the other hand, both the original PAP and the newly introduced HPAP show negligible drifts in energy (0.004 and 0.015 kcal·(mol^−1^·ps^−1^), respectively) over the duration of the 25-ps simulations. The root mean squared deviations of the energy over the timeseries were almost the same for HPAP (~0.82 kcal·mol^−1^) and PAP (~0.76 kcal·mol^−1^). 

## 4. Discussion

**Figure 7:**
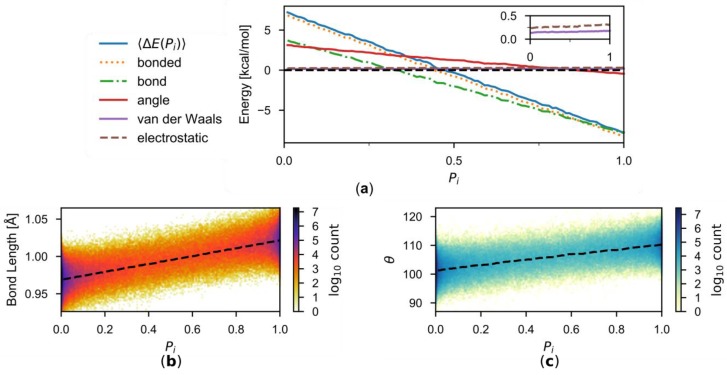
(**a**) Decomposition of $\langle \Delta E\left(P_{i}\right)\rangle$ into various energy components. The inset shows the van der Waals and electrostatic components in greater detail. (**b**) The distribution of O-H bond as functions of $P_{i}$. The average is indicated by the dashed curve. (**c**) Same as (**b**), but for H-O-H angle.

It is interesting to note that the correction $\langle \Delta E\left(P_{i}\right)\rangle$ is approximately a linear function of $P_{i}$ in the present work. To understand the origin of this approximate linearity, let us consider a given *i*-th buffer group. When the description of this buffer groups is switched between the two employed MM force fields, SPC/Fw and TIP3P/Fs, there will be changes in the non-bonded (van der Waals and electrostatic) interactions through which this buffer group interacts with active-zone groups, environmental-zone groups, and the other buffer groups. Also changing are the intramolecular bonded (O-H bond and H-O-H angle) interactions within this buffer group. The decomposition of $\langle \Delta E_{i}\left(P_{i}\right)\rangle$ is depicted in Figure 7a according to

(20a) $$\langle \Delta E_{i}\rangle \;=\;\langle V_{i=\mathrm{SPC}/\mathrm{FW}\;}-V_{i=\mathrm{TIP}3P/\mathrm{Fs}}\rangle$$

(20b) $$\;=\;\langle \Delta E_{i,\mathrm{bonded}\;}\rangle +\langle \Delta E_{i,\mathrm{nonbonded}}\rangle \;=\;\langle \Delta E_{i,\mathrm{bond}}\rangle +\langle \Delta E_{i,\mathrm{angle}}\rangle +\langle \Delta E_{i,\mathrm{vdw}}\rangle +\langle \Delta E_{i,\mathrm{elec}}\rangle$$

where *i* denotes the *i*-th buffer group. 

The bonded energy terms are the dominant contributors to $\Delta E_{i}$. This is not surprising because of the similarity in the nonbonded interaction parameters (Table 1). Both the O-H bond and H-O-H angle energies are sizeable, but the O-H bond energy is overall more significant. The difference in the O-H bond energies between the two descriptions are

(21) $$\;\Delta E_{\mathrm{bond}\;}\;=\;k_{1}{\left(x-x_{01}\right)}^{2}-k_{2}{\left(x-x_{02}\right)}^{2}$$

where *x* is the instantaneous O-H bond length, and $k_{1}$ and $k_{2}$ are the force constants parameters and $x_{01}$ and $x_{02}$ the equilibrium geometric parameters of the two water models, respectively. If $k_{1}\;=\;k_{2}\;=\;k$, as it is for the two water models employed here, one has

(22) $$\;\Delta E_{\mathrm{bond}\;}\;=\;-2k\left(x_{01}-x_{02}\right)x+k\left(x_{01}^{2}-x_{02}^{2}\right)$$

This means that the energy difference will vary linearly with respect to the instantaneous bond length. Figure 7b suggests smooth structural changes of the water molecule in the buffer zone between the TIP3P/Fs model at $P_{i}\;=\;0$ and the SPC/Fw model at $P_{i}\;=\;1$ and confirms that the average instantaneous O-H bond length in the simulations is indeed approximately linear with respect to $P_{i}$. The situation is similar in the H-O-H angle energy (Figure 7c), where $k_{1}\approx k_{2}\;=\;k$, and the linearity also roughly holds. As a result, $\langle \Delta E\left(P_{i}\right)\rangle$ is approximately linear with respect to $P_{i}$.

Of course, the above analysis can only be conducted when the interactions are pairwise, which is true here for the dual-MM simulations. However, because pairwise interactions are usually predominant (e.g., accounting for ~80% of total interaction energies in water [60]), the approximate linearity may still hold when models with higher-order many-body potentials are employed, albeit to a less extent, unless the two employed potentials differ significantly from each other. In reality, the potentials should agree with each other reasonably well in the buffer region, otherwise it is likely that at least one potential is very inaccurate and should not be used at all. 

We note that while the forces by the correction term cancel the average transition forces, the cancellation is not exact at every step. Inexact transient cancellations lead to “residue” forces, whose effects on the simulation may or may not lead to erroneous solvation structures, which probably varies from case to case. While further investigations will be needed to explore this in the future, it is conceivable that narrow, symmetric distributions of the residue forces can help to minimize their effects, which is the case in this work. Therefore, it is prudent to match the potentials as closely as possible in the buffer zone. At this point, we note that Jiang et al. [61] are developing MM water models specifically designed for adaptive QM/MM simulations.

Overall, the results presented here demonstrate that the HPAP can both yield accurate solvation structures and conserve energy in *NVE* simulations. The progress thus fills a gap in the PAP algorithms. The successful applications of the HAMBC treatment to both SAP by Boreboom et al. [42] and PAP in this work suggest that other distance-based adaptive QM/MM schemes may also benefit from this treatment. We expect that the new HPAP algorithm will be useful to many applications where simulations of Hamiltonian systems are required. Future studies are especially encouraged to investigate the cases where multiple types of buffer groups (e.g., water and ions) are present and to explore the treatments for inhomogeneous systems (e.g., ion channels).

## Figures and Tables

**Table 1:** Force field parameters for the water models employed in this work.

Interaction	Parameter	TIP3P/Fs ^1^	SPC/Fw ^2^
O-H stretching	*k*_b_ [kcal/mol/Å^2^]	1059.162	1059.162
	*B*_0_ [Å]	0.960	1.012
H-O-H bending	*k*_θ_ [kcal/mol/rad^2^]	68.097	75.900
	*θ*_0_ [deg]	104.500	113.24
Electrostatic	*q*_H_ [e]	0.417	0.41
	*q*_O_ [e]	−0.834	−0.82
van der Waals (O only)	*R*_min_/2 [Å]	1.7682	1.7767
	*ε* [kcal/mol]	−0.1522	−0.1524

^1^ Ref. [58] ^2^ Refs. [57,58].
